# Biomechanical assessments of high-fluence corneal cross-linking with supplemental oxygen using optical coherence elastography in *ex vivo* rabbit eyes

**DOI:** 10.1117/1.JBO.31.9.095002

**Published:** 2026-09-09

**Authors:** Vance Thompson, Kenneth A. Beckman, Rajesh Rajpal, Behrouz Tavakol, Stuart Elmhurst, David B. Usher, Jason M. Hill, Tomas Navratil

**Affiliations:** a Vance Thompson Vision, Sioux Falls, South Dakota, United States; b Comprehensive Eyecare of Central Ohio, Westerville, Ohio, United States; c See Clearly Vision Group, McLean, Virginia, United States; d Glaukos Corporation, Burlington, Massachusetts, United States; e Glaukos Corporation, Aliso Viejo, California, United States

**Keywords:** corneal cross-linking, epithelium-on, supplemental oxygen, optical coherence elastography, increased corneal stiffness

## Abstract

**Significance:**

Epithelium-off corneal collagen cross-linking (CXL) has drawbacks related to epithelial removal. Earlier epithelium-on approaches without supplemental oxygen showed limited efficacy due to inadequate permeation of the photoenhancer and oxygen into the corneal stroma. Improved epithelium-on CXL with improved photoenhancer permeation and supplemental oxygen has demonstrated effective cross-linking in two phase 3 trials.

**Aim:**

The study aims to evaluate the effect of supplemental oxygen during epithelium-on CXL in an *ex vivo* animal study mirroring the clinical CXL procedure, using an in-house built optical coherence elastography (OCE) system that measures biomechanical changes (stiffness) in the cornea.

**Approach:**

Epithelium-on CXL was performed on two groups of *ex vivo* rabbit eyes at a UV fluence of $10\;J/{\mathrm{cm}}^{2}$ and irradiance of $30\;\mathrm{mW}/{\mathrm{cm}}^{2}$. Eyes in Group 1 were treated under normoxic (ambient air, 21% oxygen) conditions, whereas eyes in Group 2 were treated under hyperoxic (>90% oxygen) conditions.

**Results:**

Forty eyes treated under hyperoxic conditions and pulsed illumination exhibited a statistically significant greater increase (p<0.0001) in shear modulus (81% larger increase), as a measure of stiffness, compared with eyes treated under normoxic conditions.

**Conclusions:**

These results strongly suggest that the use of supplemental oxygen during epithelium-on CXL produces stronger inter-corneal collagen cross-links and greatly improves corneal stiffness, potentially lessening or preventing the risk of vision loss for patients with keratoconus.

## 1 Introduction

Keratoconus is a type of corneal ectasia in which progressive thinning and irregular steepening of the cornea result in a distinct cone-shaped topography. This disease affects ∼1.4 per 1000 people worldwide, although the disease prevalence varies greatly between different populations.^1^^,^^2^ It may originate from genetic and/or environmental factors.^2^^–^^4^ Keratoconus results in symptoms such as blurred or distorted vision, which interfere with a patient’s quality of life and lead to blindness if allowed to progress without treatment.^3^^,^^5^^,^^6^ A variety of symptomatic treatments have been developed, including different types of contact lenses and intracorneal ring segment (ICRS) implantation,^3^ but these provide short-term relief and cannot prevent disease progression because they fail to target the underlying corneal biomechanical changes seen in keratoconus. In severe cases, a corneal transplant is needed to prevent further disease progression, but has the potential risk of graft rejection.^7^ Therefore, the corneal collagen cross-linking procedure was developed to offer a safe and effective solution to this dilemma by inducing a photochemical reaction between oxygen, photoenhancer (riboflavin), and ultraviolet A (UVA) light to strengthen weakening collagen fibers in the corneal stroma. Corneal cross-linking is now considered the gold standard for keratoconus treatment.^8^^–^^10^ This procedure has greatly reduced the need for corneal transplants and other treatment regimens, ultimately reducing the disease and economic burdens while creating a breakthrough in treatment for keratoconus patients across the globe.^9^^,^^11^

Despite the demonstrated ability of corneal collagen cross-linking to produce an advancement in patient care, the traditional epithelium-off protocol requires removal of the corneal epithelium and takes at least one hour to complete.^8^ This procedure may cause painful side effects associated with the removal of the corneal epithelium, along with an increased risk of infection, epithelial defect, bandage contact lens use, and a prolonged period of healing.^12^^–^^14^ In addition, the nature of treatment may be intolerable for certain patients.^15^ As a result, the epithelium-on corneal collagen cross-linking procedure with supplemental oxygen, in which there is no removal of the corneal epithelium, was designed and validated in clinical trials.^16^^,^^17^ The addition of permeability enhancers such as benzalkonium chloride (BAC) to the riboflavin formulation allows sufficient absorption of riboflavin through the epithelial barrier and its distribution into the corneal stroma.^18^ Shorter accelerated corneal collagen cross-linking procedures were derived from the Bunsen–Roscoe Law of Reciprocity, which implies that the same effect of the photochemical corneal collagen cross-linking reaction can be produced by increasing the UVA irradiation while decreasing the duration of treatment, as long as the same total energy is applied.^19^ Some studies conducted without supplemental oxygen have suggested that accelerated epithelium-on corneal collagen cross-linking is less effective than the standard epithelium-off protocol.^20^^–^^22^ However, multiple authors have argued that this could be due to a lack of total applicability of the Bunsen–Roscoe Law of Reciprocity to corneal collagen cross-linking, perhaps since the reaction also relies on oxygen rather than just UVA and riboflavin.^19^^,^^23^^,^^24^ Therefore, multiple follow-up studies have been conducted with the goal of improving the efficacy of corneal collagen cross-linking administered with shorter treatment durations.^25^^,^^26^

**Fig. 1:**
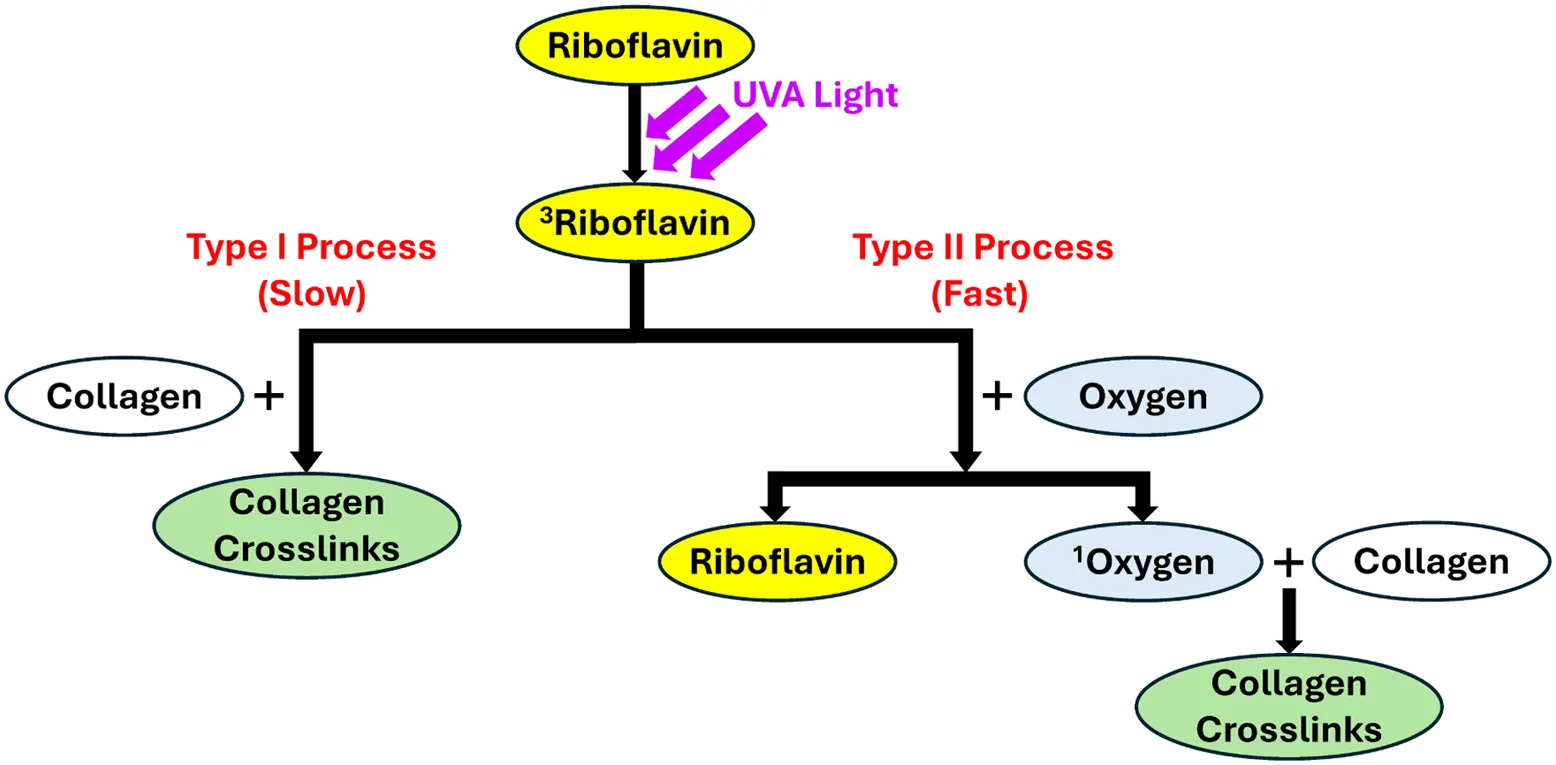
UVA light activates riboflavin (^3^Riboflavin) which leads to collagen cross-linking via two pathways: Type I (slow) without oxygen and Type II (fast) with activated singlet oxygen (^1^Oxygen).

Although the rate-limiting factor of the corneal collagen cross-linking reaction remains debated, growing evidence suggests that oxygen availability is the primary constraint, owing to rapid stromal oxygen depletion that exceeds the rate of replenishment under ambient (room-air) conditions.^17^^,^^23^^,^^27^^,^^28^ During corneal collagen cross-linking, riboflavin reacts with UVA light and is converted from an excited singlet molecule to an excited triplet form.^29^ Subsequently, either a Type I or Type II reaction occurs to create the collagen cross-links (Fig. 1). When oxygen is present, the faster and more efficient Type II reaction occurs in which the excited state of riboflavin reacts with triplet oxygen to form singlet oxygen, which is reactive and therefore short-lived. This reactive oxygen species rapidly produces collagen cross-links formed preferentially between side chains of aromatic amino acid residues in the collagen molecules. In the absence of oxygen, the slower, less efficient Type I reaction predominates, under which the excited state of riboflavin reacts directly but slowly with collagen molecules.^17^^,^^18^^,^^29^^,^^30^

**Fig. 2:**
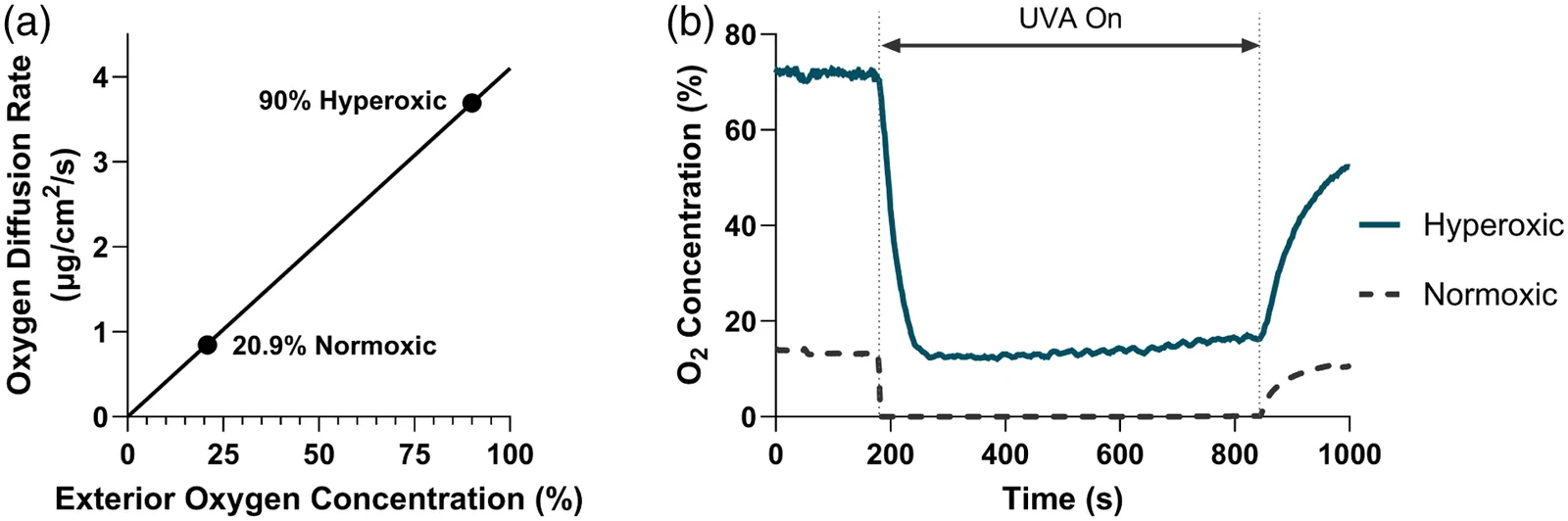
(a) Rate of oxygen flow across the anterior surface of the cornea is linearly dependent on the oxygen concentration at the anterior surface. Changing from normoxic to hyperoxic conditions increases the rate of oxygen replenishment in the stroma by greater than four-fold. (b) Intrastromal oxygen concentration before, during, and after UVA irradiation under normoxic (dashed line) and hyperoxic (blue solid line) conditions with an intact corneal epithelium.

Importantly, Kamaev et al.^27^ and Seiler et al.^23^ demonstrated that oxygen is quickly depleted at high UVA irradiances, thus impeding the corneal collagen cross-linking reaction and thereby creating a rationale for oxygen supplementation during the clinical cross-linking procedure. In support of this rationale, we previously demonstrated that supplemental oxygen during corneal collagen cross-linking mitigates rapid oxygen depletion during UVA irradiation and enhances the efficiency of the photochemical cross-linking reaction, leading to significantly improved corneal stiffening and flattening^31^ (Fig. 2).

The goal of our current *ex vivo* study was to examine the importance of supplemental oxygen in the epithelium-on cross-linking procedure as determined by direct measurement of corneal stiffness using optical coherence elastography (OCE) which provides a quantitative, noninvasive approach to directly measure corneal stiffness and thereby evaluate corneal tissue health and detect abnormalities.^32^ This technology incorporates optical coherence tomography (OCT) to detect motion in response to mechanical loading.^33^^–^^35^ Through its unique imaging capabilities, OCE spatially maps the stress-strain response with high resolution and sensitivity to analyze tissue excitation and quantify biomechanical properties such as shear modulus as a measure of stiffness.^33^ In the context of keratoconus, OCE is a valuable tool for exploring how corneal collagen cross-linking can be optimized to create additional collagen cross-links in the cornea, which in turn increases corneal stiffness, thereby helping to prevent vision loss.^36^^,^^37^

In this head-to-head comparison, cross-linking was conducted in the presence and absence of supplemental oxygen during the epithelium-on procedure with OCE to assess the biomechanical properties of corneal tissues. We are primarily interested in the relative differences between increases in corneal stiffness as a result of the epithelium-on procedure in the absence or presence of supplemental oxygen. Measurements of corneal stiffness can be sensitive to the experimental methodology and testing conditions, particularly in *ex vivo* samples. Different excitation methodologies and frequencies can stimulate distinct wave modes and propagation velocities on the surface of the cornea and within corneal tissue. Previous studies have demonstrated velocity–dispersion relationships for various symmetric and asymmetric guided Lamb-wave modes in *ex vivo* cornea.^38^^–^^40^ Some models have been refined to incorporate factors such as small variations in central corneal thickness or fluid-to-solid interaction at the posterior corneal surfaceconditions that more accurately reflect physiological boundaries.^41^ These corrections are critical for improving absolute estimates of biomechanical parameters and for enabling comparison across studies. However, these corrections are often specific to particular experimental setups and may not be generalizable to all experimental setups. Thickness-related corrections often treat hydration-induced changes as purely geometric, without accounting for concurrent alterations in tissue material properties. In *ex vivo* corneal samples, changes in hydration can simultaneously affect corneal thickness and intrinsic biomechanics, making it difficult to differentiate whether observed differences in wave behavior arise from geometry, material properties, or both. Minimizing hydration-related variations in corneal thickness during experimentation therefore provides a more robust approach, allowing wave propagation to be analyzed under stable conditions and enabling the use of a simplified wave propagation model.

To address these challenges, we employed an experimental strategy allowing the use of a simplified model. Specifically, we used a single-frequency (2 kHz) harmonic excitation source, which enabled a clean, well-isolated antisymmetric guided wave mode with a single propagation velocity in *ex vivo* rabbit corneas. We implemented custom hydration chambers to help minimize any changes in corneal thickness during experiments due to hydration, thus ensuring stable geometric conditions and material biomedical properties of the corneas throughout the scans. In addition, “pre-treatment” OCE scans, which served as the baseline for each eye were collected after riboflavin administration and immediately before UVA irradiation, thereby further eliminating potential variability associated with corneal thickness changes during drug loading. In addition, our study design used paired conditions, keeping all testing, scanning, and processing parameters identical between groups except for the hypothesis variable presence or absence of supplemental oxygen. Together, these controls enabled us to rely on the simplified relationship $G\propto \;c^{2}$ – where G is corneal shear modulus, and c is the shear wave velocity and we report relative stiffness changes, rather than absolute values, for statistical comparisons with high confidence.

## 2 Methods

### 2.1 Ex Vivo Experiments

The objective of this *ex vivo* animal study was to evaluate the effect of supplemental oxygen on the efficacy of epithelium-on corneal collagen cross-linking. The study included two experimental groups using paired *ex vivo* rabbit eyes. All experimental conditions were kept identical between groups, except for one variable: Group 1 eyes were treated with epithelium-on corneal collagen cross-linking under normoxic conditions (ambient-level oxygen, 21%), whereas eyes in Group 2 were treated under hyperoxic conditions (oxygen concentration >90%).

**Fig. 3:**
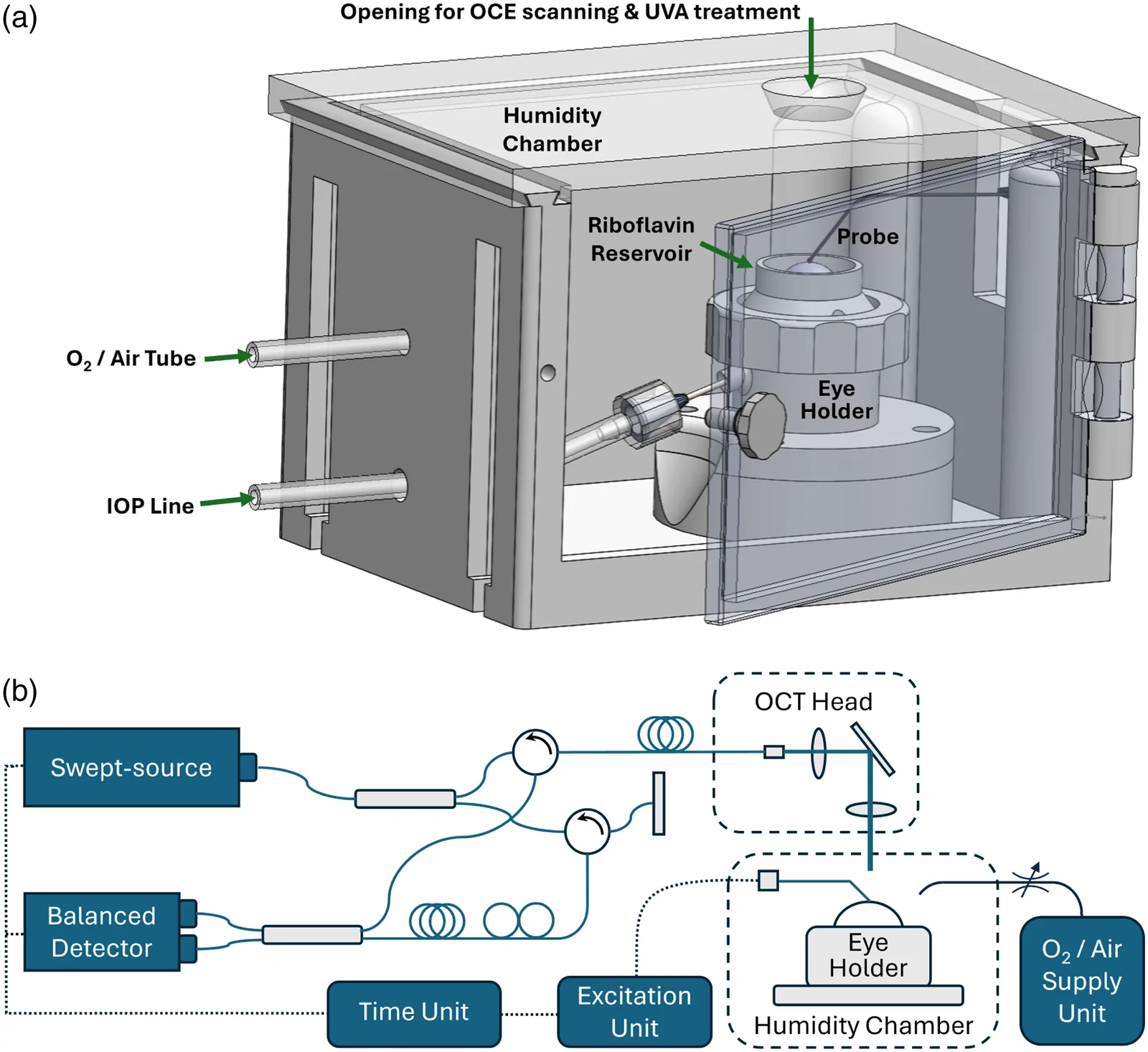
Experimental setup for *ex vivo* OCE measurements. (a) Schematics of the custom humidity chamber and eye holder assembly. The chamber housed the eye holder and riboflavin reservoir ring while maintaining hydration and constant IOP. The OCT imaging path and UVA beam passed through an open window in the chamber, and the OCE excitation probe was introduced through a side opening. (b) Schematic of the in-house built OCE system, including the swept-source OCT system, mechanical excitation probe, and synchronization/control hardware. The OCT head and UVA crosslinking device remained outside the humidity chamber and were positioned over the chamber only during their respective scanning or treatment steps.

New Zealand White Rabbit Eyes (Pel-Freez Biologicals, Rogers, Arkansas, United States) were delivered in medium and tested the day after postmortem. Each pair was stabilized to room temperature while still in medium. Prior to experimentation, both eyes in each pair were inspected for epithelial and corneal integrity—visually first, and subsequently by OCT upon being mounted on the eye holders. Pairs of eyes with defects were excluded. Only eyes with an intact epithelium in good condition were included for testing. A total of 40 eyes were used. Each eye was mounted on a custom-built eye holder positioned within a custom-designed humidity chamber to reduce dehydration and maintain intraocular pressure (IOP) near 15 mmHg via an elevated IV bag. The humidity chamber was fabricated by 3D printing using clear and white resin materials on a Formlabs Form 4 printer. The riboflavin reservoir ring was similarly custom-designed and 3D printed using white resin. The eyeholders ensured a fixed, upward-facing orientation. Two such humidity chambers allowed near-simultaneous testing of each eye in a pair. A schematic of the humidity chamber, eye holder, and other compartments is provided in Fig. 3 to illustrate the experimental configuration.

Two different clinically available isotonic riboflavin solutions were applied to the intact cornea using a reservoir on the anterior surface of the epithelium, using a 3D-printed ring mounted on the eye holder. These solutions, provided by Glaukos, were applied in two stages: Part 1 (0.25% riboflavin-5’-phosphate with 0.02% BAC) for 4 min and Part 2 (0.22% riboflavin-5′-phosphate) for 6 min. One eye of each pair (Group 2) received >90% oxygen inside the chamber, whereas the contralateral eye (Group 1) received ambient air (21% oxygen) at an identical flow rate.

Two custom UVA devices (Glaukos, Burlington, Massachusetts, United States) were used to treat all eyes by delivering a UVA fluence of $10\;J/{\mathrm{cm}}^{2}$ at an irradiance of $30\;\mathrm{mW}/{\mathrm{cm}}^{2}$, using 1 s on: 1 s off pulsed illumination for a total exposure time of 11 min and 6 s. To minimize bias, we randomized the order of eye pair testing and the assignment of treatment protocols to chambers. Experiments were conducted across four separate batches of rabbit eyes to further control for inter-batch variability.

### 2.2 OCE Scanning and Data Processing

Corneal biomechanical changes were assessed using an in-house built OCE system (Fig. 3), incorporating a phase-stabilized swept-source OCT (ATR210, Thorlabs, Newton, New Jersey, United States) and a piezoelectric actuator to deliver mechanical excitation to the corneal apex at a fixed frequency of 2 kHz. The OCT system operated at a center wavelength of 1060 nm with a fixed A-scan rate of 100 kHz, ∼100  dB sensitivity, an axial resolution of 8  μm in water, and an imaging depth of 10 mm. For each M-mode acquisition, 250 A-lines were collected at each lateral position. The excitation and OCT acquisition were synchronized using an FPGA module on a National Instruments RIO chassis (NI9147). The OCT head was mounted on a linear rail to allow positioning over each humidity chamber during OCE scanning.

**Fig. 4:**
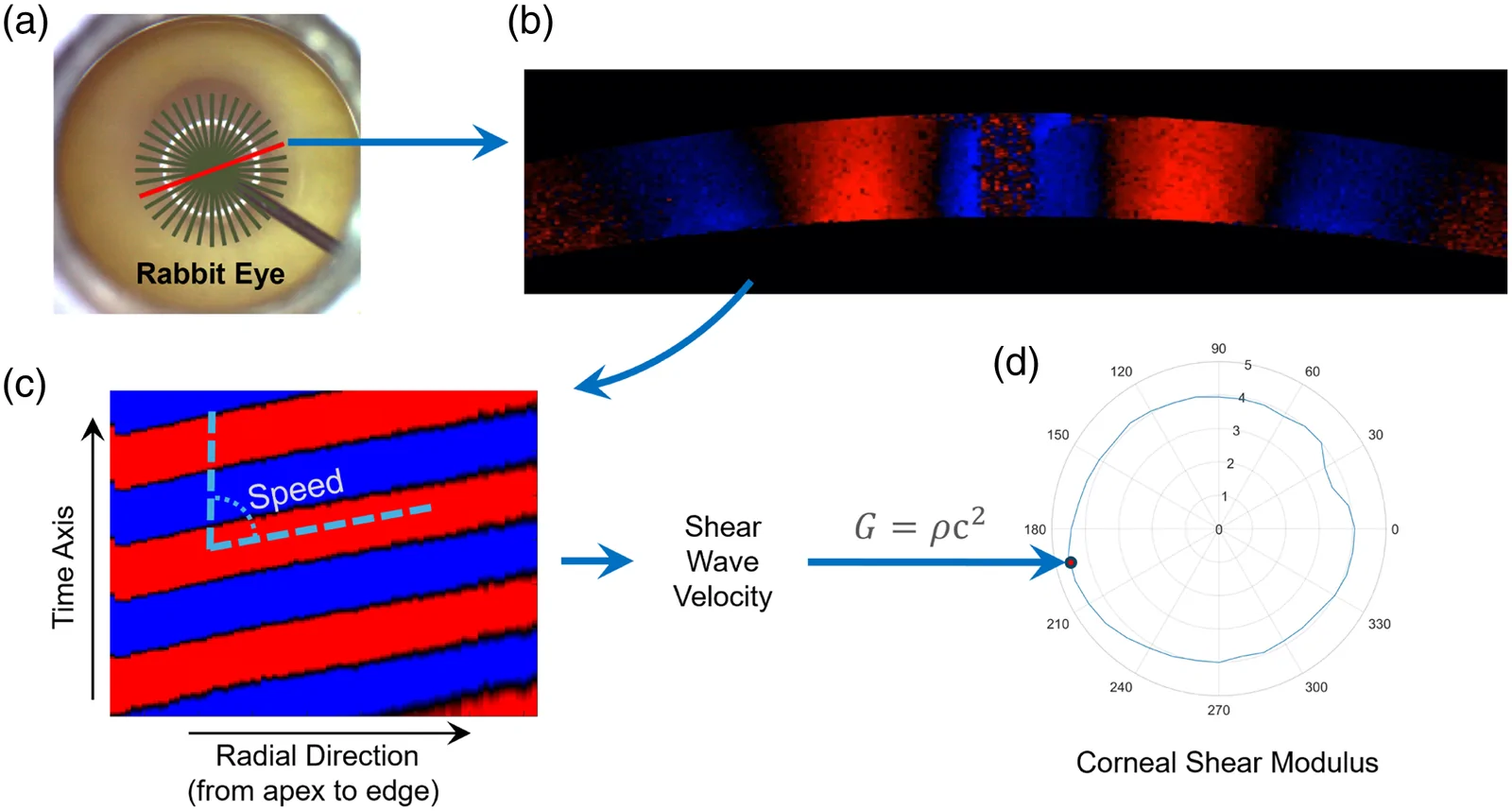
Steps to calculate corneal shear modulus from an OCE scan. (a) One OCE scan includes 18 meridians. (b) Axial displacements for different time points are calculated for each meridian. (c) A 2D axial displacement map in the time-space domain is constructed for each half-meridian. (d) Each point on the polar plot represents the corneal shear modulus calculated for a half-meridian. Red/blue coloring schematically illustrates the sign and spatial pattern of vertical displacement and is not intended as a calibrated displacement magnitude scale.

For each OCE scan, M-scans were acquired along 18 meridians, corresponding to 36 half-meridians [Fig. 4(a)]. The process for deriving corneal shear modulus from OCE data has been described in detail in previous publications;^33^^,^^38^^,^^42^ here, we briefly summarize the method used. The axial displacement is computed from phase changes in the A-scan signals [Fig. 4(b)]. For each meridian, the anterior corneal surface was first identified from the OCT B-scan, and axial displacement was calculated within the anterior stromal region. The central region within ∼500  μm of the apex was excluded from the displacement analysis. To improve signal robustness, the vertical displacement was averaged over approximately the anterior 200  μm beneath the corneal surface at each lateral position. The resulting displacement profile was then used to construct the spatiotemporal displacement map shown in Fig. 4(c). In this representation, the radial axis corresponds to the lateral position along the scanned meridian in the OCT image. A 2D fast Fourier transform (FFT) is applied to this displacement map to extract the dominant wavenumber at the excitation frequency, from which the shear wave velocity is determined. The shear modulus G for each half-meridian is estimated using the simplified relation $G=\rho c^{2}$, where ρ is the corneal density, assumed to be $1035\;\mathrm{kg}/m^{3}$, and c is the calculated shear wave velocity. Figure 4(d) presents a polar plot of the shear modulus measured across all half-meridians for a representative eye.

OCE scans were collected immediately before and immediately after UVA irradiation. The use of the humidity chamber, along with pre- and post-treatment scanning, allowed for isolated measurement of cross-linking-induced stiffening while minimizing confounding effects from dehydration. For each timepoint, we collected 2 OCE scans within 30 s. Corneal thickness near the apex was measured from OCT B-scan images acquired as part of the OCE scans before and after treatment. A paired Student’s t-test (conducted in GraphPad Prism) was used to assess the statistical significance of differences in corneal thickness and shear modulus changes between the two groups.

## 3 Results

Corneal thickness near the apex, measured from OCT B-scan images acquired during OCE scans, was 587±53  μm before treatment and 578±50  μm after treatment. The eyes experienced an average corneal thickness change of only −1.38%±1.33% between the pre- and post-treatment OCE scans. There was also no significant difference in thickness change between the two groups (p=0.69).

**Fig. 5:**
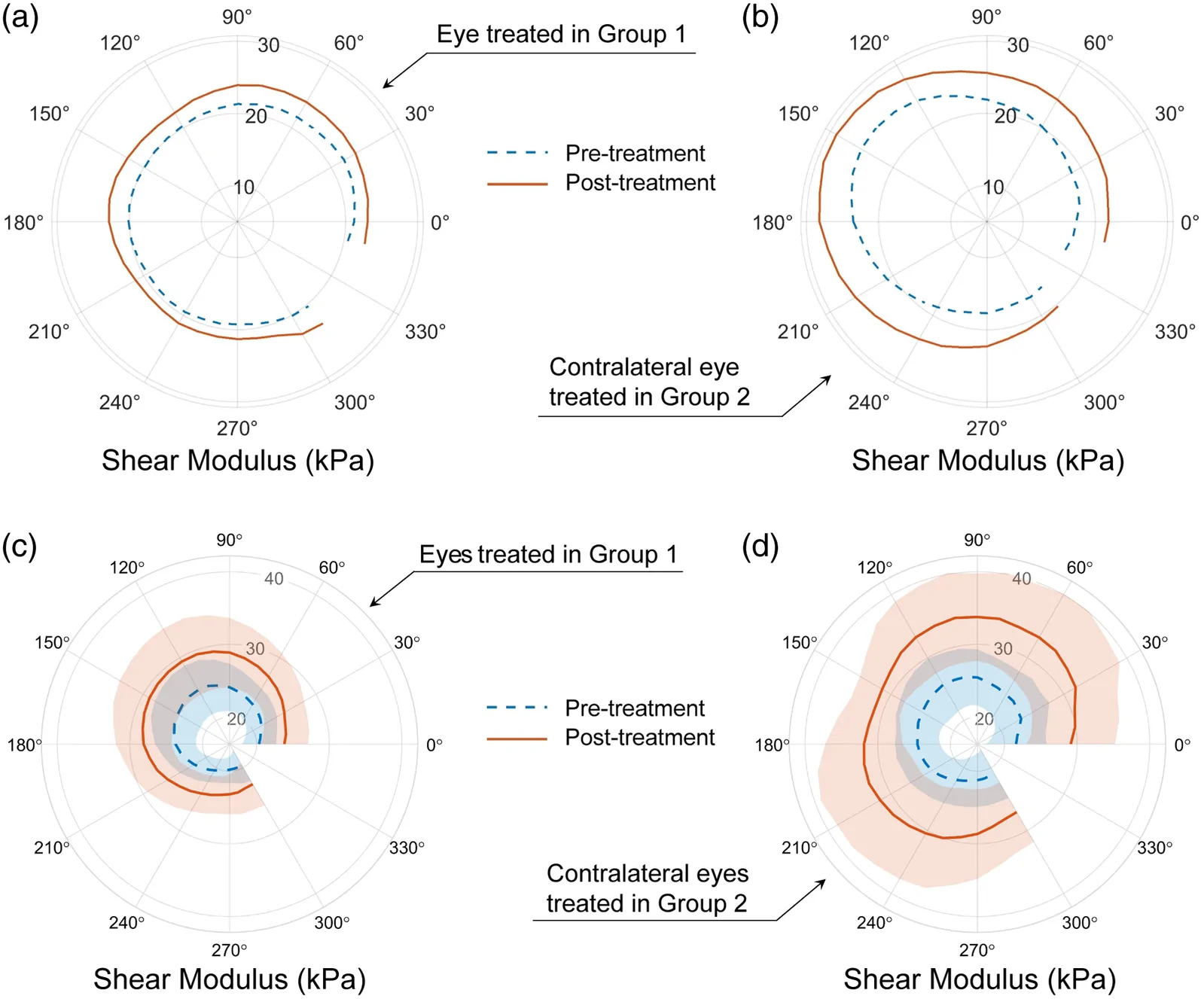
Angularly resolved corneal shear modulus measurements pre- and post-treatment. (a) and (b) Representative paired-eye example showing an eye treated under normoxic conditions (Group 1) and the contralateral eye treated under hyperoxic conditions (Group 2). (c) and (d) Group-level angularly resolved shear modulus distribution across all eyes in Group 1 and their contralateral eyes in Group 2, shown to summarize the spatial distribution of stiffness changes across the cohort. Solid lines indicate the group means, and shaded regions indicate ±1 standard deviation. The 0 deg position denotes the experimental reference orientation used during OCE scanning and does not correspond to a confirmed anatomical landmark.

Corneal shear moduli measured across different meridians for a representative pair of eyes; one treated under hyperoxic conditions and the other under normoxic conditions are shown in Fig. 5. A small angular range (315 deg to 345 deg) could not be scanned due to occlusion by the excitation probe.

**Fig. 6:**
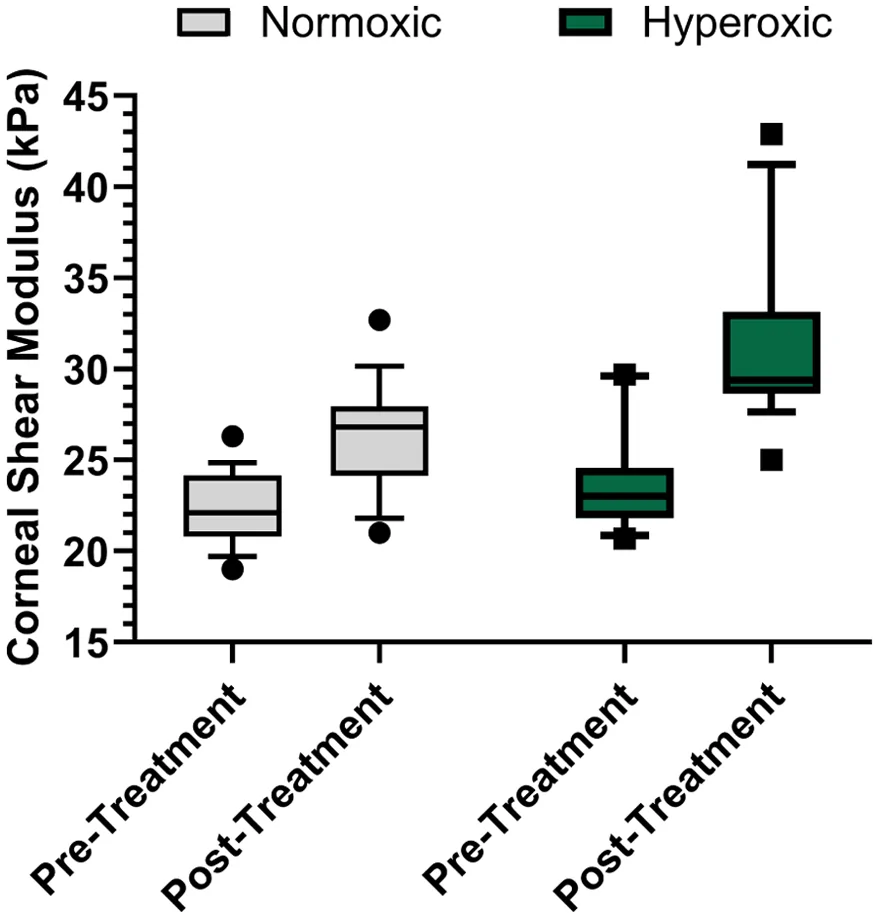
Corneal shear modulus was measured before and after cross-linking on eyes treated under normoxic conditions in Group 1 (left) and their contralateral eyes treated under hyperoxic conditions in Group 2 (right). Boxes indicate the 25th to 75th percentiles, the horizontal line indicates the median, and whiskers extend from the 10th to 90th percentiles.

To summarize and compare changes across eyes, Fig. 6 presents the median shear modulus values across meridians, before and after treatment. Both groups showed a significant increase in corneal stiffness following the corneal collagen cross-linking procedure. However, the increase was statistically superior and more pronounced in eyes treated under hyperoxic conditions.

**Fig. 7:**
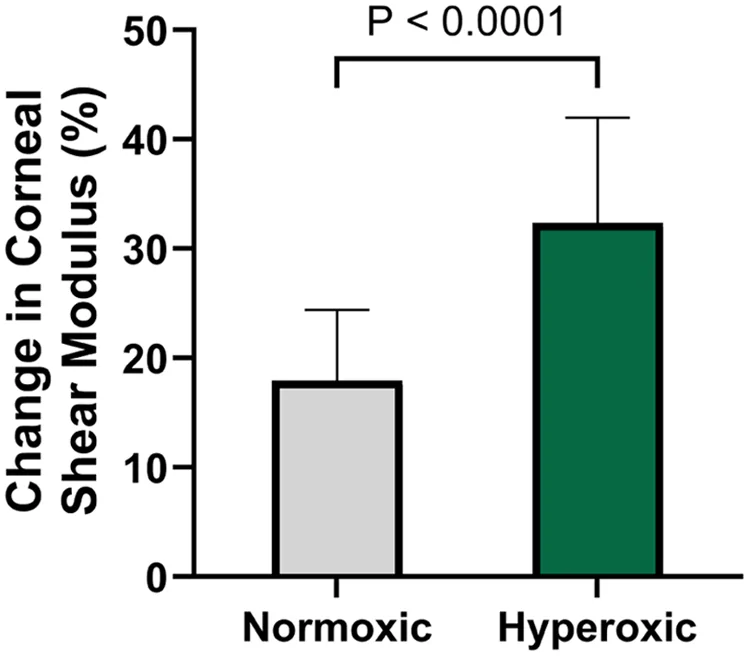
Changes in corneal shear modulus after corneal collagen cross-linking for eyes treated in Group 1 (normoxic conditions) and their contralateral eyes treated in Group 2 (hyperoxic conditions).

To quantify this difference, we computed the percentage change in shear modulus averaged over meridians for each eye using the formula: Percentage Change=(Post-treatment−Pre-treatment)/Pre-treatment×100. The results are shown in Fig. 7. The pairwise percentage change in shear modulus of each pair was used in the statistical test. The data passed both the Shapiro–Wilk (p=0.72) and Anderson–Darling (p=0.83) normality tests.

Eyes treated under supplemental oxygen (Group 2, hyperoxic conditions) exhibited an 81% larger increase in shear modulus (confidence interval: 54% to 116%) compared with eyes treated under ambient oxygen level (Group 1, normoxic conditions) (p<0.0001).

These findings demonstrate that supplemental oxygen enhances the biomechanical efficacy of high-fluence epithelium-on corneal collagen cross-linking, supporting the inclusion of supplemental oxygen for improved keratoconus treatment outcomes.

## 4 Discussion

Epithelium-on oxygen-enriched corneal collagen cross-linking, which combines epithelial penetration enhancement to optimize stromal riboflavin saturation and supplemental oxygen to maintain sufficient oxygen concentration in the corneal stroma, represents a transformative treatment option for patients with keratoconus, as it directly addresses two major limitations of traditional epithelium-off cross-linking. It uses an incision-free method, which mitigates harmful side effects and extended healing and visual recovery associated with the removal of the corneal epithelium while reducing procedure time by utilizing higher irradiances. The results of our study demonstrate that epithelium-on corneal collagen cross-linking with supplemental oxygen is effective at improving corneal biomechanical stiffness. This outcome was demonstrated through an 81% increase in shear modulus for corneas treated under hyperoxic (>90% oxygen) conditions compared with those treated under normoxic (ambient air, 21% oxygen) conditions. These results are in line with our previous work^31^ and are consistent with other studies that also compared the biomechanical properties induced by corneal collagen cross-linking treatment under various oxygen conditions. For instance, Richoz et al.^43^ illustrated that porcine corneas treated with epithelium-off corneal collagen cross-linking under normoxic conditions (21% oxygen) had a significantly higher Young’s moduli compared with corneas treated under low-oxygen (<0.1% oxygen) conditions, inducing a greater effect of stiffness and elasticity. Similarly, Wang et al.^44^ and Alkhalde et al.^45^ exemplified that oxygen-enriched and intracameral oxygenated epithelium-off corneal cross-linking respectively, produced significantly greater stiffening effects compared with standard epithelium-off corneal cross-linking in porcine eyes. Wang et al.^46^ also studied the effects of accelerated epithelium-off corneal collagen crosslinking *in vivo* and *ex vivo*, under normoxic and hyperoxic conditions in rabbit corneas. The authors discovered that corneas treated under hyperoxic conditions displayed significantly greater resistance to corneal deformation *in vivo*. In addition, *ex vivo* corneas treated under hyperoxic conditions had significantly higher elasticity in the nasal-temporal directions compared with the control at applied stresses of 0.15 and 0.20 MPa. Therefore, each of these studies demonstrates that the presence of oxygen is crucial for successful corneal cross-linking. Consequently, supplemental oxygen plays an exceptionally vital role in epithelium-on corneal cross-linking procedures by increasing their efficiency and effectiveness. This overrides the limitation of low permeability from preserved corneal epithelium.

The results of our study also highlight the potential of OCE as a noninvasive and highly sensitive method for assessing biomechanical changes in the cornea, primarily in a nonclinical setting. Although still relatively new, OCE has emerged as a valuable tool for evaluating corneal biomechanics following corneal cross-linking, with prior studies confirming that corneal collagen cross-linking increases corneal stiffness.^37^^,^^47^^,^^48^ Another distinct advantage of OCE is its ability to detect corneal anisotropy, or the direction-dependent mechanical behavior of the cornea, enabling more sensitive detection of localized stiffness changes than is possible with traditional destructive biomechanical techniques such as uniaxial and biaxial tension testing.^37^^,^^39^^,^^49^^,^^50^

Although the present analysis summarized meridian-level measurements to support paired statistical comparisons, OCE provides spatially resolved biomechanical information that may be used in future studies to evaluate regional variations in corneal response following cross-linking.

Beyond the advantages of the OCE technique itself, several methodological strengths of this study helped minimize bias and confounding variables. These include the paired-eye study design, randomization of eye pair testing, timely OCE scanning before and after riboflavin administration and UVA irradiation, maintenance of IOP near physiological levels, restriction to samples with intact epithelium and corneal integrity, and the use of humidity chambers to reduce dehydration. The small and statistically indistinguishable changes in corneal thickness observed between pre- and post-treatment scans indicate that hydration-related effects on corneal thickness were minimal. This approach allowed the use of a simplified wave propagation model and reduced uncertainty in the interpretation of wave-based stiffness measurements. As a result, relative changes in corneal stiffness could be attributed with greater confidence to the treatment effect rather than experimental artifacts.

In the present study, baseline OCE measurements were acquired after riboflavin administration and immediately before UVA exposure to isolate the biomechanical effects of cross-linking and enable direct comparison between normoxic and hyperoxic treatment conditions. Future studies may incorporate OCE measurements acquired prior to riboflavin administration to further characterize the individual contributions of riboflavin loading and hydration changes to corneal biomechanical responses. In addition, future studies may investigate any possible temporal evolution of corneal biomechanical changes following cross-linking by incorporating OCE measurements at later post-treatment time points, while carefully controlling tissue hydration and viability in *ex vivo* conditions.

Although the findings are promising, it is important to acknowledge that the *ex vivo* nature of the study may limit its direct translation to human corneal behavior following corneal collagen cross-linking in the clinic. In addition, the use of healthy rabbit eyes may not fully capture the biomechanical response of human keratoconic corneas. Nevertheless, given the controlled two-arm design in which normoxic and hyperoxic conditions were the only differing variable we believe the positive effects observed with supplemental oxygen in high-fluence, epithelium-on corneal collagen cross-linking are likely to translate similarly to patients with keratoconus.

## 5 Conclusion

Supplemental oxygen used to increase oxygen concentration in the corneal stroma, in combination with an epithelial penetration enhancer to optimize stromal riboflavin saturation, has been shown to significantly increase the effectiveness of epithelium-on corneal collagen cross-linking as demonstrated by increased corneal stiffness when compared with epithelium-on corneal collagen cross-linking without supplemental oxygen. Moreover, the OCE methodology used in this study is a promising technology that can be used to quantify corneal stiffness induced during corneal collagen cross-linking. Therefore, epithelium-on corneal collagen cross-linking with oxygen supplementation provides a potentially transformative approach for keratoconus management and patient care.

## Data Availability

The data are available from the corresponding author upon reasonable request.
